# Role of O-C2 angle in the development of dysphagia in patients with halo-vest fixation

**DOI:** 10.1186/s12891-020-3155-2

**Published:** 2020-02-28

**Authors:** Midori Miyagi, Hiroshi Takahashi, Kazuaki Tsuchiya, Hideki Sekiya, Satoru Ebihara

**Affiliations:** 1 Department of Rehabilitation Medicine, Toho University Graduate School of Medicine, 6-11-1 Omori-nishi, Tokyo, Ota-ku 143-8541 Japan; 2 Department of Orthopedic Surgery, Toho University Omori Medical Center, Tokyo, Japan; 3 Department of Oral Surgery, Toho University Omori Medical Center, Tokyo, Japan

**Keywords:** Halo-vest, Dysphagia, O-C2 angle, Food intake level

## Abstract

**Background:**

Dysphagia is one of the most serious complications in patients treated with a halo-vest brace. However, the cause of dysphagia development by halo-vest fixation is not yet clear. We therefore investigated the incidence of dysphagia and cervical alignment as well as clinical data from medical charts in patients treated with a halo-vest brace.

**Methods:**

We retrospectively reviewed clinical data from the medical charts of 49 patients who had undergone halo-vest fixation. Occipito (O)-C2 angle, C2-C6 angle, and pharyngeal inlet angle were assessed by lateral plain X-rays of the cervical spine. The impacts of these parameters on incidence and severity of dysphagia were analyzed.

**Results:**

Thirteen patients (32%) suffered from dysphagia during halo-vest fixation, and age and length of intensive care unit (ICU) stay were greater in the dysphagia group (*p* = 0.044 and 0.013, respectively) than in those who did not develop dysphagia. O-C2 angle was smaller in the dysphagia group (*p* = 0.016). After multivariate logistic analysis, body mass index, ICU stay, and O-C2 angle remained as independent risk factors related to incidence of dysphagia. Spearman rank correlation showed a negative correlation between ICU stay and Food Intake Level Scale (FILS) (*p* = 0.026), and a positive correlation between O-C2 angle and FILS (*p* = 0.008).

**Conclusion:**

This study suggested that O-C2 angle is related to both incidence and severity of dysphagia due to halo-vest fixation.

## Background

As a part of the treatment course for cervical spine fracture and/or spinal cord injury, cervical orthoses are required to immobilize cervical vertebrae. Among cervical orthoses, the halo-vest brace provides the greatest fixation of the head and neck [1, 2]. The halo-vest has been a common tool for immobilization of the cervical spine since its development in 1959 by Perry and Nickel [3]. However, complications such as pin loosening and infection have been reported in patients fixed using the halo-vest brace [2].

Among the complications with the halo-vest brace, dysphagia is one of the most serious, leading to life-threatening aspiration pneumonia, particularly in elderly patients [2, 4]. Morishima et al. [5] found that halo-vest fixation with unnatural alignment caused dysphagia in normal, healthy volunteers. Bradley et al. [6] found that a longer stay in the intensive care unit (ICU) was associated with the incidence and severity of dysphagia in traumatically injured patients treated with a halo-vest brace. Although they analyzed various factors from medical charts, including severity of injury, length of ICU stay, duration of ventilator use and length of hospital stay, the status of cervical alignment was not evaluated in that study.

In patients who have undergone cervical fusion, accumulating evidence suggests that cervical alignment provides a predictor of postoperative dysphagia [7–18]. With occipitocervical fusion, the decrease in O-C2 angle is a predictor of dysphagia after surgery [7–16]. The decrease in O-C2 angle shifts the mandible posteriorly with the tongue root, resulting in a reduction in the pharyngeal space. This backward movement of the tongue root presumably causes oropharyngeal stenosis and impairment of adequate motion of the epiglottis. In addition, Kaneyama et al. [19] recently reported the pharyngeal inlet angle (PIA), which also correlates with pharyngeal space, as a predictor of dysphagia in patients who underwent occipitocervical fusion.

On the other hand, in cervical fusion not involving the cranium, the C2-C7 angle is reported to be related to dysphagia after surgery [17, 18]. Tian et al. [18] reported that deviation from cervical lordosis causes posterior pharyngeal wall bulging, resulting in a reduction in the pharyngeal space.

Taken together, we hypothesized that not only clinical data from medical charts such as length of ICU stay and mechanical ventilation, but also un-optimized cervical alignment would impact on the incidence of dysphagia in patients with halo-vest fixation. The aim of this study was to elucidate the relationship between cervical alignment and incidence of dysphagia in patients treated with a halo-vest brace by evaluating lateral radiological findings of the cervical spine, as well as patient clinical data from medical charts. Relationships between extracted risk factors and severity of dysphagia were also analyzed.

## Methods

### Subjects

We retrospectively reviewed the medical charts of patients who had undergone halo-vest fixation in our institute between January 2006 and August 2016. Collected clinical data included demographic information and duration of wearing the halo-vest, length of ICU stay, duration of ventilator use, presence or absence of tracheostomy, etiology, presence or absence of surgery for cervical fixation before halo-vest fixation, and complications. Patients with severe dementia (Mini-Mental State Examination score < 10), facial trauma, neurodegenerative disease, obvious cerebral infraction, spinal cord injury of grade A or B according to the Frankel classification, or confused state (Richmond Agitation-Sedation Scale below − 2 or above + 2) throughout the period of wearing the halo-vest were excluded from this study.

### Dysphagia evaluation

Severity of dysphagia was assessed by Food Intake Level Scale (FILS) from medical chart descriptions of the eating condition of patients [20]. In our institution, patients with FILS level 8–10 are followed by nurses who belong to each ward, and patients with FILS level 1–7 are followed by the institutional team for dysphagia rehabilitation and are appropriately assessed by fiberoptic endoscopic and/or video-fluoroscopic swallow studies. Severity of dysphagia was evaluated within 1 week after halo-vest fixation in a sitting position as far as possible. Based on FILS level, patients were classified into non-dysphagia (FILS level: 10) and dysphagia (FILS level: 1–9) groups. FILS is defined as follows [20]: Level 1, no swallowing training; Level 2, swallowing training not using food; Level 3, swallowing training using a small quantity of food; Level 4, easy-to-swallow food less than the quantity of a meal, but predominantly with alternative nutrition; Level 5, easy-to-swallow food orally ingested for 1–2 meals, but alternative nutrition also given; Level 6, easy-to-swallow food for 3 meals, but alternative nutrition used; Level 7, easy-to-swallow food orally ingested in 3 meals with no alternative nutrition given; Level 8, patient eats three meals, only excluding food that is particularly difficult to swallow; Level 9, no dietary restriction, and patient ingesting three meals orally, with medical considerations; and Level 10, normal.

### Radiographic measurements

We reviewed lateral plain X-rays of the cervical spine under conditions of halo-vest fixation to measure O-C2 angle, C2-C6 angle, narrowest oropharyngeal airway space (nPAS) as an indicator of pharyngeal space [14], and pharyngeal inlet angle (PIA) [19] within 2 days from FILS evaluation.

**Fig. 1:**
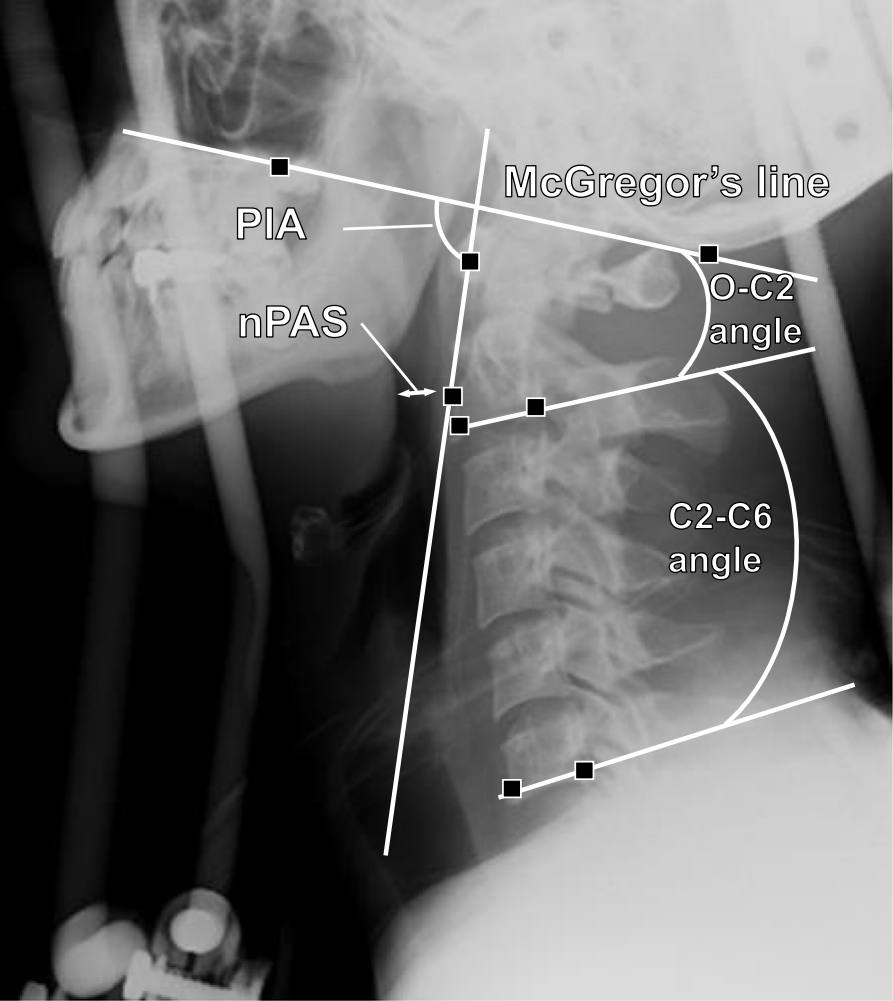
Representative lateral plain X-rays of the cervical spine. O-C2 angle is defined as the angle between McGregor’s line (the line connecting the posterior edge of the hard palate to the most caudal portion of the occipital curve) and the inferior endplate line of C2. C2-C6 angle is defined as the angle between the inferior endplate line of C2 and the inferior endplate line of C6. The narrowest anteroposterior distance of the oropharynx between the tips of the uvula and epiglottis (double white arrow) is used as the nPAS. PIA represents the angle between McGregor’s line and the line made by drawing connecting the center of the C1 anterior arch to the apex of the cervical sagittal curvature

O-C2 angle was defined as the angle between McGregor’s line (made by drawing a line connecting the posterior edge of the hard palate to the most caudal portion of the occipital curve) and the inferior endplate line of C2. C2-C6 angle was defined as the angle between the inferior endplate line of C2 and the inferior endplate line of C6. Positive values for both O-C2 and C2–C6 angles indicated lordosis at the measured segments [14]. The definition of nPAS was the narrowest anteroposterior distance of the oropharynx between the tips of the uvula and epiglottis [14]. Finally, PIA was defined as the angle between McGregor’s line and the line made by drawing a line connecting the center of the C1 anterior arch to the apex of the cervical sagittal curvature [19] (Fig. 1).

We also estimated soft-tissue swelling in the prevertebral area on lateral plain X-rays of the cervical spine. According to a study by Rojas et al., which showed the normal range of thickness of prevertebral soft tissues, we regarded thicknesses greater than 6 mm at C2, 7 mm at C3, or 18 mm at C6 on lateral plain X-rays as representing prevertebral soft-tissue swellings [21].

We reviewed the presence or absence of odontoid fracture, and the type of odontoid fracture as classified by Anderson and D’Alonzo [22]. Type II C2 fractures carry a high risk of nonunion, and thus may affect functional outcomes in patients with halo-vest fixation [23].

### Statistical analysis

Values are expressed as mean ± standard deviation and range. Differences in baseline characteristics were tested using the two-tailed t test for continuous variables, the Mann-Whitney *U* test for non-normally distributed variables, and the χ^2^ test for categorical variables. Multivariate logistic analyses with forward selection based on likelihood ratio were used to evaluate the association between incidence of dysphagia and age, body mass index (BMI), ICU stay, and O-C2 angle, which were selected as independent variables showing values of *P* < 0.1 in the two-tailed *t* test, χ^2^ test, and Mann-Whitney test in Table 1 (Model 1).

**Table 1:** Comparison of clinical data from medical charts

Clinical data from medical charts	Non-dysphagia (*n* = 28)	Dysphagia (*n* = 13)	*p* value
Age (years)	54.6 ± 20.4 (13 to 78)	69.2 ± 13.02 (49 to 91)	0.044^a*^
Female/male	12/16	5/8	0.790^b^
Body mass index (kg/m^2^)	23.6 ± 4.6 (15.6 to 32.2)	20.9 ± 2.4 (14.7 to 24.0)	0.055^a^
Wearing duration (days)	57.1 ± 25.9 (14 to 111)	55.5 ± 24.8 (15 to 108)	0.856^a^
ICU stay (days)	0.21 ± 0.81 (0 to 4)	1.38 ± 2.1 (0 to 6)	0.013^c*^
Ventilator day (days)	1.0 ± 1.67 (0 to 7)	3.46 ± 6.4 (0 to 20)	0.236^c^
Tracheostomy (n)	1	2	0.232^b^
Cervical fusion before halo-vest fixation (n)	8	2	0.308^b^
Prevertebral soft tissue swelling (n)	9	2	0.231^b^
Etiology			
Trauma (n)	16	8	0.790^b^
OPLL (n)	3	0	0.539 ^b^
Cervical spondylosis (n)	3	2	0.645 ^b^
Suppurative spondylitis (n)	2	2	0.579 ^b^
Other (n)	4	1	1.00 ^b^
Complications			
Hypertension (n)	6	6	0.146 ^b^
Diabetes (n)	4	3	0.659 ^b^

Values represent mean ± standard deviation (range in parentheses)

*OPLL* ossification of posterior longitudinal ligament

^a^ Two-tailed *t* test; ^b^ χ^2^ test; ^c^ Mann-Whitney test

^*^ Statistically significant

Moreover, the presence or absence of surgery for cervical fixation before halo-vest fixation, soft-tissue swelling in the prevertebral area, and days on ventilation, which were possible confounding factors for the incidence of dysphagia, were included as variables in addition to the variables in Model 1 for multivariate logistic analyses (Model 2).

Spearman correlation coefficients were used to evaluate whether FILS level correlated with each independent factor identified in multivariate logistic analyses, and nPAS. Moreover, Pearson correlation coefficients were used to evaluate whether O-C2 angle correlated with C2-C6 angle, nPAS, or PIA. Values of *P* < 0.05 were considered statistically significant. We used SPSS version 17.0 software (SPSS, Chicago, IL) for all analyses.

## Results

A total of 49 patients underwent halo-vest fixation in our institute between January 2006 and August 2016. Eight patients were excluded from this study: 5 patients suffered from spinal cord injury classed as Grade A or B according to the Frankel classification; and 1 patient failed to show C2-C6 on lateral plain X-rays due to obesity; and 2 patients suffered from facial trauma. Data from a total of 41 patients were analyzed.

For the total cohort, age was 59.2 ± 19.6 years, height was 160.2 ± 9.9 cm, weight was 58.5 ± 13.9 kg, BMI was 22.7 ± 4.2 kg/m^2^, duration of wearing the halo-vest was 56.5 ± 25.6 days, length of ICU stay was 0.59 ± 1.46 days, duration of ventilator use was 1.78 ± 4.02 days, 10 patients underwent cervical fusion before halo-vest fixation, and 11 patients showed prevertebral soft-tissue swelling. Etiology of halo-vest fixation was trauma (*n* = 24), cervical spondylosis (*n* = 5), suppurative spondylitis (*n* = 4), ossification of the posterior longitudinal ligament (*n* = 3) and other conditions (*n* = 5; 2 patients with rheumatoid arthritis, 1 patient with idiopathic cervical dislocation, 1 patient with metastatic carcinoma, and 1 patient with attempted suicide by hanging).

Based on FILS scoring, 13 patients (32%) suffered dysphagia during halo-vest fixation. Comparisons of clinical data from medical charts between non-dysphagia and dysphagia groups are shown in Table 1. No significant differences in sex, duration of wearing the halo-vest, duration of ventilator use, proportion of patients with tracheostomy, proportion of patients with cervical fusion before halo-vest fixation, proportion of patients with prevertebral soft-tissue swelling, the reason for halo-vest fixation, and complications were seen between non-dysphagia and dysphagia groups. However, age and length of ICU stay were significantly greater in the dysphagia group than in the non-dysphagia group (*p* = 0.044, 0.013, respectively). BMI tended to be greater in the dysphagia group than in the non-dysphagia group (*p* = 0.055).

Twelve patients (29%) were diagnosed with odontoid fracture, comprising Anderson and D’Alonzo type II in 3 patients, and type III in 9 patients [22]. No difference in type of odontoid fracture was seen between non-dysphagia and dysphagia groups.

In radiographic data for all subjects, O-C2 angle was 16.4 ± 9.5°, C2-C6 angle was 13.0 ± 10.7°, nPAS was 13.5 ± 5.6 mm and PIA was 91.1 ± 8.5°. Comparisons of radiographic measurements between non-dysphagia and dysphagia groups are shown in Table 2. O-C2 angle was significantly smaller in the dysphagia group than in the non-dysphagia group. On the other hand, C2-C6 angle, nPAS and PIA did not differ significantly between groups.

**Table 2:** Comparison of radiographic measurements

Measurement	Non-dysphagia (*n* = 28)	Dysphagia (*n* = 13)	*p* value
O-C2 angle (°)	18.8 ± 8.0	11.2 ± 10.28	0.016^a^
C2-C6 angle (°)	13.9 ± 11.0	11.2 ± 9.6	0.465
nPAS (mm)	14.2 ± 4.8	12.2 ± 6.6	0.296
PIA (°)	91.8 ± 9.0	89.8 ± 7.2	0.499

Values represent mean ± standard deviation

*nPAS* narrowest oropharyngeal airway space, *PIA* pharyngeal inlet angle

^a^ Statistically significant

Results of multivariate logistic analyses using age, BMI, ICU stay, and O-C2 angle as covariates (Model 1) are shown in Table 3. BMI (odds ratio [OR] 0.59, 95% confidence interval [CI] 0.38–0.93, *p* = 0.024), ICU stay (OR 3.68, 95%CI 1.27–10.62, *p* = 0.016), and O-C2 angle (OR 0.83, 95%CI 0.71–0.97, *p =* 0.021) remained independent risk factors related to incidence of dysphagia.

**Table 3:** Multivariate logistic analysis for risk factors of dysphagia

	OR	95%CI	*p* value
BMI (kg/m^2^)	0.52	0.297–0.899	0.020^a^
ICU stay (days)	4.93	1.387–17.490	0.014^a^
O-C2 angle (°)	0.79	0.650–0.949	0.012^a^

*OR* odds ratio, *CI* confidence interval

^a^ Statistically significant

Moreover, in the logistic analyses, we added presence or absence of operation for cervical fixation before halo-vest fixation, soft-tissue swelling in the prevertebral area, and days on ventilation as covariates, as these were also possible confounding factors for the incidence of dysphagia (Model 2). However, no differences in statistical results were found between Models 1 and 2.

**Fig. 2:**
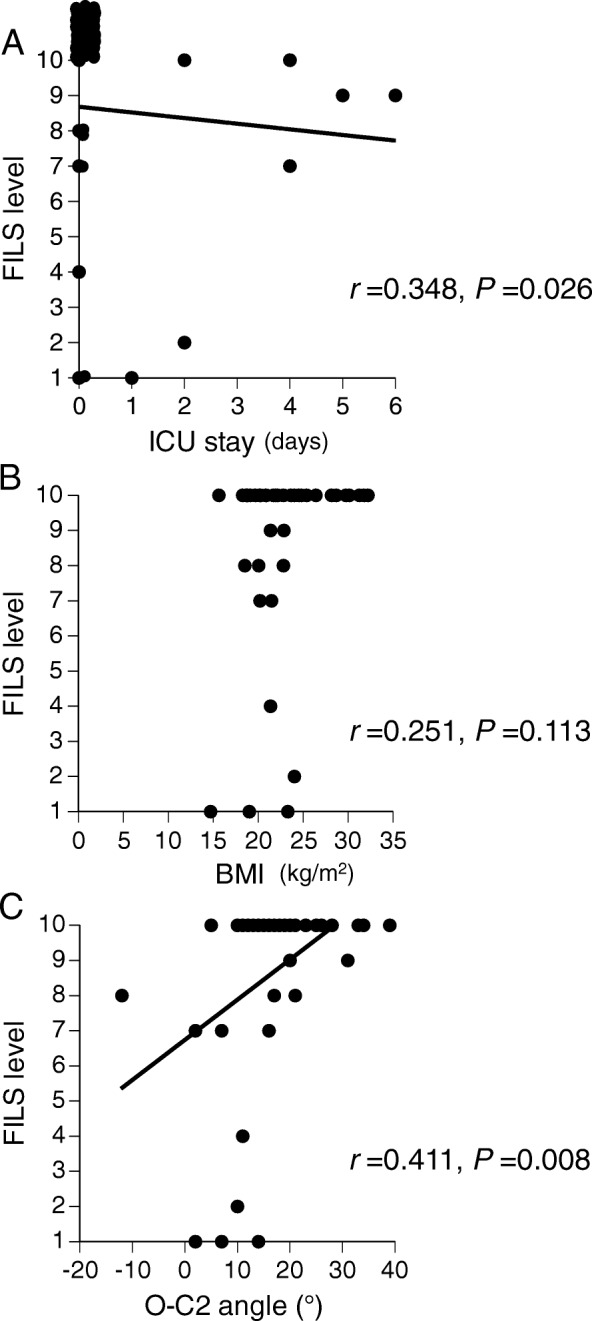
**a** Scatter diagram showing association of ICU stay and FILS level. **b** Scatter diagram showing association of BMI and FILS level. **c** Scatter diagram showing association of O-C2 angle and FILS level

A negative correlation was seen between ICU stay and FILS level (*r* = 0.348, *p* = 0.026; Fig. 2a). No significant correlation was seen between BMI and FILS level (Fig. 2b). A positive correlation was seen between O-C2 angle and FILS level (*r* = 0.411, *p* = 0.008; Fig. 2c). No significant correlation was seen between nPAS and FILS level (*r* = 0.272, *p* = 0.085) (data not shown).

**Fig. 3:**
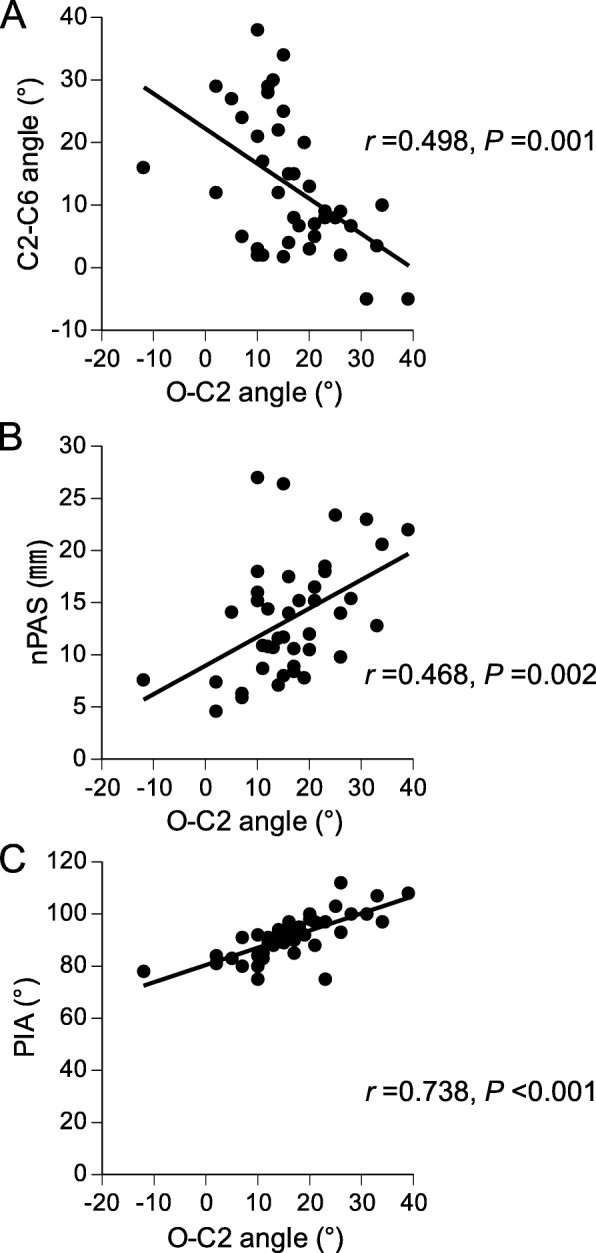
**a** Scatter diagram showing association of O-C2 and C2-C6 angles. **b** Scatter diagram showing association of O-C2 angle and nPAS. **c** Scatter diagram showing association of O-C2 angle and PIA

A negative correlation was seen between O-C2 and C2-C6 angles (*r* = 0.498, *p* = 0,001; Fig. 3a). A positive correlation was seen between O-C2 angle and nPAS (*r* = 0.468, *p* = 0.002; Fig. 3b). A positive correlation was also seen between O-C2 angle and PIA (*r* = 0.738, *p* < 0.001; Fig. 3c). The coefficient ratio (η) for incidence of dysphagia was 0.373, 0.117, 0.167, and 0.109 for O-C2 angle, C2-C6 angle, nPAS, and PIA, respectively.

## Discussion

Although previous studies have identified dysphagia as a complication of halo-vest fixation, cervical alignment has not been investigated as a cause of dysphagia in patients treated with a halo-vest brace. The present study identified ICU stay, BMI, and O-C2 angle as independent risk factors for the incidence of dysphagia among patients with halo-vest fixation. Furthermore, we found that both smaller O-C2 angle and longer ICU stay were associated with lower FILS level.

We reviewed 41 patients who had undergone halo-vest fixation. The mean age of all patients was 59.2 years, older than patients in previous studies (range, 28.3–43 years). The proportion of male subjects in our study was 59%, consistent with previous studies [24–26]. In terms of causative factors, 59% of patients showed a traumatic etiology. Using clinical data from medical charts, we found that age and duration of ICU stay were greater in the dysphagia group than in the non-dysphagia group.

Bradley et al. [6] retrospectively reviewed 56 patients treated with a halo-vest due to traumatic cervical fracture. In that study, the rate of dysphagia was much higher (66%) than in our study (32%), presumably because our subjects included non-traumatic patients. The proportion of males in their dysphagia group (65%) was consistent with our observations (62%). The study showed that ICU stay was longer in patients with dysphagia and was associated with severity of dysphagia, consistent with our findings.

We revealed O-C2 angle as a factor independently associated with the incidence of dysphagia among patients with halo-vest fixation, and smaller O-C2 angle was related to lower FILS level. On the other hand, C2-C6 angle did not differ significantly between groups. Previous studies in patients who had undergone cervical fusion involving the cranium showed that O-C2 angle was related to the incidence of dysphagia [7–16]. Since the halo-vest is most effective method of fixation for vertebrae of the upper cervical spine, halo-vest fixation may resemble cervical fusion with the cranium rather than that without the cranium.

In this study, nPAS did not differ significantly between groups, and no significant correlation was evident between nPAS and FILS level, even though a positive correlation was seen between O-C2 angle and nPAS (Fig. 3b). This result suggests that O-C2 angle may affect not only the pharyngeal space, but also other factors related to dysphagia in patients with halo-vest fixation. In addition, PIA did not differ between patients with and without dysphagia. Taken together, narrowing of the pharyngeal space may not be crucial for the development of dysphagia during halo-vest fixation. Further studies are required to elucidate the relationship between O-C2 angle and incidence of dysphagia during halo-vest fixation.

BMI did not differ significantly between groups. However, BMI remained independently associated with dysphagia among patients with halo-vest fixation according to logistic analysis. Maeda et al. [27] reviewed 224 elderly inpatients with and without dysphagia. They reported that the BMI of patients was lower in the dysphagia group than in the non-dysphagia group, consistent with our results. Since they revealed sarcopenia as an independent risk factor for dysphagia in hospitalized elderly individuals, our patients with dysphagia may have suffered from sarcopenia [27].

Figure 2c suggests several clinical issues worthy of consideration. First, the difference of 5° in O-C2 angle resulted in a difference of 1 point in the FILS score, suggesting the usefulness of describing the radiographic angle measurement compared to gaze angle or gross clinical appearance. At the time of halo-vest assembly, lateral cervical spine X-ray including the skull to ensure an adequate O-C2 angle may be helpful in finding the best position to allow normal swallowing in patients with halo-vest fixation. Previously, the recommendation for positioning in halo-vest fixation to maintain normal swallowing physiology has been simply to avoiding cervical hyperextension [5]. In this study, we revealed another important issue of positioning to prevent dysphagia in halo-vest fixation.

When we perform halo-vest fixation, we pay attention to fixing the spine in a cervical neutral position under X-ray fluoroscopic imaging. Among the 130° of total flexion-extension range of motion of the neck, only 20–30° (15–25%) is involved in O-C2 articulations [28]. The positioning therefore usually occurs through the lower cervical spine, and variance in C2–6 becomes low. O-C2 angle is not usually given much attention during the procedure for halo-vest fixation. However, we found that O-C2 angle was significant in patients with halo-vest fixation to prevent dysphagia.

Figure 3a and c show that O-C2 angle correlated significantly with both C2-C6 angle and PIA, suggesting the existence of collinearity among each of the radiographic measurement items investigated in the study. The coefficient ratios suggested that O-C2 angle may provide the greatest contribution to the incidence of dysphagia among the items examined. However, other components of occipitocervical alignment such as translation forward or back in the horizontal plane and/or angulation at different areas of cervical spine may also affect the incidence of dysphagia. Further studies are warranted to clarify other radiographic measurement items affecting swallowing functions.

This study had several limitations, including the retrospective study design. In addition, fiberoptic endoscopic and/or video-fluoroscopic swallow studies were not performed for every patient. Another limitation was the lack of radiographic data before halo-vest fixation. We were therefore unable to evaluate changes in alignment, which might be more relevant than angles at a single time point.

## Conclusions

In this study, O-C2 angle was significantly smaller in patients who developed dysphagia during halo-vest fixation. BMI, ICU stay, and O-C2 angle were independent risk factors for dysphagia in patients with halo-vest fixation. Moreover, smaller O-C2 angle and longer ICU stay were associated with lower FILS. These results suggest that inadequate extension at O-C2 may contribute to swallowing difficulties in patients with halo-vest fixation.

## Data Availability

The datasets used and/or analyzed during current study are available from the corresponding author on reasonable request.

## Abbreviations

BMI: Body mass index
CI: Confidence interval
FILS: Food intake level scale
ICU: Intensive care unit
nPAS: Narrowest oropharyngeal airway space
O-C2 angle: Occipito-C2 angle
OPLL: Ossification of posterior longitudinal ligament
OR: Odds ratio
PIA: Pharyngeal inlet angle
